# Giant liver hemangioma with adult Kasabach-Merritt syndrome: Case report and literature review

**DOI:** 10.1097/MD.0000000000007688

**Published:** 2017-08-04

**Authors:** Xiaolei Liu, Zhiying Yang, Haidong Tan, Li Xu, Yongliang Sun, Shuang Si, Liguo Liu, Wenying Zhou, Jia Huang

**Affiliations:** Department of Hepatobiliary Surgery, China-Japan Friendship Hospital, Beijing, China.

**Keywords:** enucleation, Kasabach-Merritt syndrome, liver hemangioma, liver resection

## Abstract

**Rationale:**

Adult Kasabach-Merritt syndrome associated with giant liver hemangioma is rare; to date, most reports have been single-case reports, and no multi-case reports or literature reviews are available.

**Diagnoses:**

We conducted a retrospective analysis of 5 cases of adult Kasabach-Merritt syndrome associated with giant liver hemangioma treated at our hospital between 2011 and 2016. All 5 patients had varying severities of leukopenia, anemia, thrombocytopenia, prolonged prothrombin time, and hypofibrinogenemia.

**Interventions:**

All the patients underwent surgery: 2 patients had left hemihepatectomy; 1 had enucleation; 1 had a right hemihepatectomy; and 1 had a left trisectionectomy.

**Outcomes:**

The 5 patients had an average operative time of 6.9 hours and an average blood loss of 3200 mL. One patient developed a biliary fistula (grade II) after the operation. There was no mortality among 5 patients. The white blood cell counts, hemoglobin, platelets, and prothrombin times of all 5 patients returned to normal after the operation. To date, a total of 11 cases of adult Kasabach-Merritt syndrome associated with giant liver hemangioma have been reported, of which 8 patients underwent surgery, and their platelets and coagulation returned to normal after the operation.

**Lessons:**

Adult Kasabach-Merritt syndrome associated with giant liver hemangioma is uncommon, and surgical treatment is risky. However, resection of the tumor corrected the abnormalities in hematological and coagulative systems.

## 1 Introduction

A hemangioma is the most common congenital benign liver tumor and is more common in women, with an estimated incidence of 0.4% to 20% in the population.^[1]^ Most clinical cases of liver hemangioma present no obvious symptoms. Once the tumor is greater than 5 cm, some patients experience abdominal discomfort or pain, but few have coagulation disorders associated with hemangiomas. A giant liver hemangioma, although uncommon, may cause serious coagulation disorders, such as Kasabach-Merritt syndrome, which presents as hemolytic anemia, thrombocytopenia, prolonged prothrombin time, and hypofibrinogenemia. Kasabach-Merritt syndrome associated with giant liver hemangioma is more common in children than in adults, and most reports of adult cases are single-case reports. Currently, the primary treatments for this condition are liver resection, enucleation, and liver transplantation. Coagulation usually returns to normal after the operation. Herein, we report 5 cases of adult Kasabach-Merritt syndrome associated with giant liver hemangioma and perform a literature review of relevant cases.

## 2 Case report

A total of 5 cases of adult Kasabach-Merritt syndrome associated with giant liver hemangioma were treated at our hospital between June 2011 and January 2017. The diagnostic criteria for Kasabach-Merritt syndrome were thrombocytopenia, anemia, hypofibrinogenemia, and a prolonged prothrombin time. Patient information was collected, including age, gender, and symptoms. Laboratory tests included blood tests, liver enzymes, and coagulation tests, whereas imaging studies focused on the size, location, number of hemangiomas, and the anatomical relationship to the surrounding vessels. The treatments and outcomes were analyzed, including the surgical procedure, operative time, amount of blood loss, amount of autologous transfusion, blood transfusion requirement, Pringle maneuver and occlusion time, morbidity, mortality, and postoperative stay. Informed consent was obtained from all patients regarding the diagnostic and therapeutic procedures. The study was approved by the Institutional Ethical Review Board of China-Japan Friendship Hospital.

**Figure 1:**
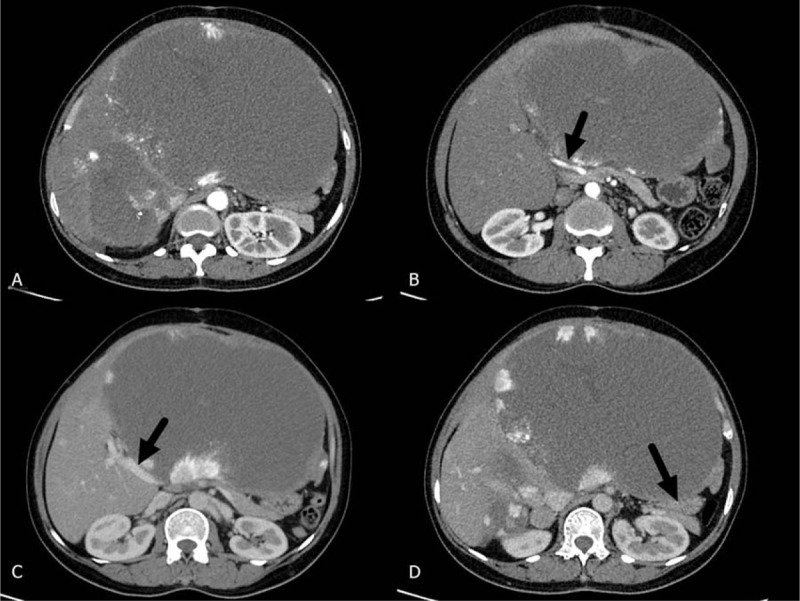
CT images of a patient with Kasabach-Merritt syndrome associated with giant liver hemangioma. (A) Multiple liver hemangiomas, with the larger one in the left liver; (B) compression of the hepatic artery (black arrow); (C) compression of the portal vein (black arrow); (D) the gastric outlet is obviously compressed (black arrow).

**Figure 2:**
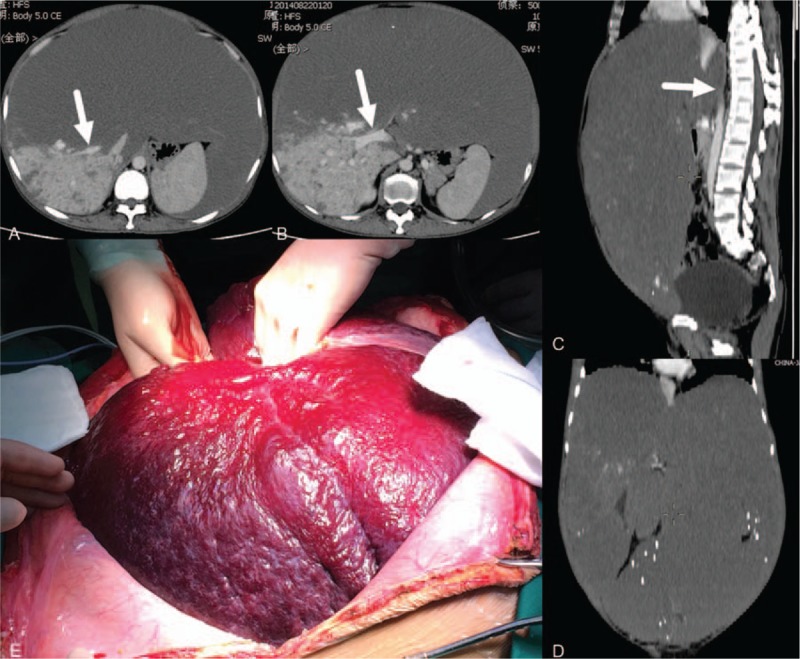
A patient with Kasabach-Merritt syndrome associated with an extremely giant liver hemangioma (50 cm). (A–D) CT images showing an extremely giant liver hemangioma, occupying the entire abdominal cavity and extending to the pelvis, with compression of the right hepatic vein (A, white arrow), right portal vein (B, white arrow), and inferior vena cava (C, white arrow); (E) intraoperative exploration revealed an extremely giant liver hemangioma occupying the entire abdominal cavity.

Among these 5 patients, there were 4 women and 1 man, with a mean age of 44.8 years (33–63 years). Presenting symptoms included abdominal distension in 3 patients and abdominal pain in 2 patients. The mean length of time from the diagnosis of hemangioma was 15.4 months (3–38 months). All 5 patients had varying severities of thrombocytopenia, anemia, neutropenia, prolonged prothrombin time, and hypofibrinogenemia. Liver enzymes were normal in all 5 patients; but 2 patients had high serum bilirubin levels (Table 1). Of the 5 patients, 4 had multiple hemangiomas, and 1 had a single hemangioma. The largest hemangioma was in the right liver in 2 cases and in the left liver in 3 cases. The hemangioma size was 31.6 cm on average (20–50 cm). Imaging studies showed gastrointestinal compression in 3 cases and compression of the first hepatic hilus in 4 cases (Fig. 1). Of these 5 patients, 2 underwent left hemihepatectomy; 1 underwent enucleation; 1 underwent right hemihepatectomy; and 1 underwent left trisectionectomy (Fig. 2). Hemangioma was confirmed pathologically after the operation in all the cases. The operative time averaged 6.9 hours (5–8.5 hours), with an average blood loss of 3200 mL (800–8000 mL). The average volume of autologous transfusion was 2000 mL (650–5800 mL) and all 5 patients had blood transfusion. Pringle maneuver was used for 3 patients to reduce the blood loss. One patient developed a biliary fistula (grade II) after the operation^[2]^ and was cured by drainage. There was no mortality among the 5 patients. The average postoperative hospital stay was 16.8 days (10–34 days). For all 5 patients, white blood cell counts, hemoglobin, platelets, and prothrombin times returned to normal after the operation (Table 2).

**Table 1:**
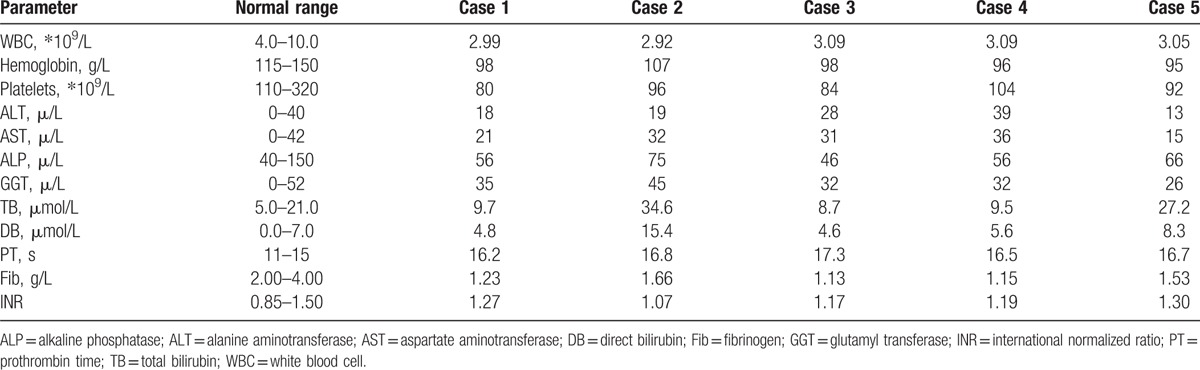
Laboratory tests of 5 patients with Kasabach-Merritt syndrome associated with giant liver hemangioma.

**Table 2:**
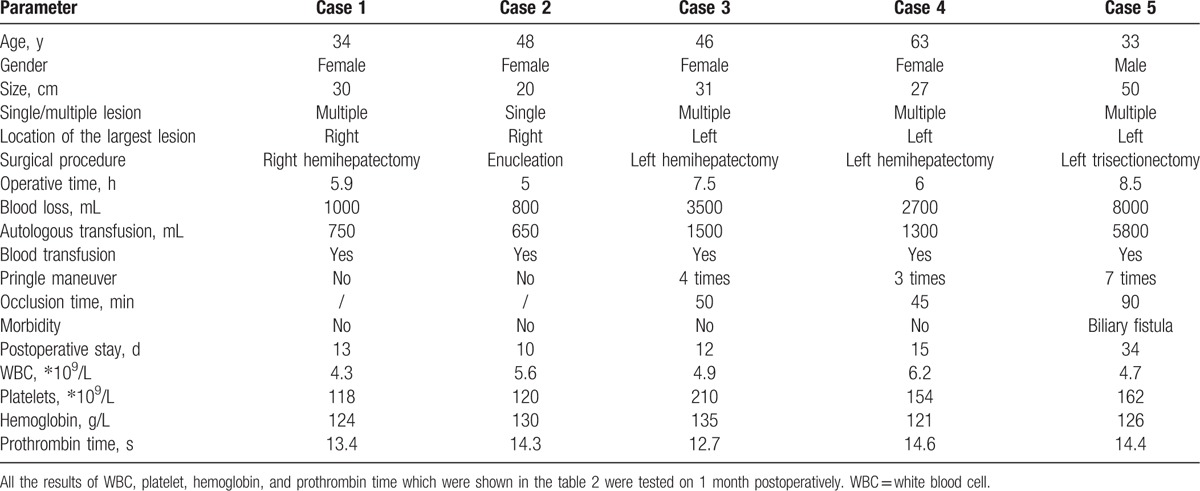
Surgical procedures and outcomes of 5 patients with Kasabach-Merritt syndrome associated with giant liver hemangioma.

## 3 Literature search and retrieval

We searched the PubMed and EMBASE databases to retrieve relevant articles published from 1996 to January 2017. The keywords used included “liver hemangioma,” “hepatic hemangioma,” and “Kasabach-Merritt syndrome.” A total of 11 articles on adult Kasabach-Merritt syndrome associated with giant liver hemangioma have been published over the past 20 years.^[3–13]^ A total of 11 cases were reported, 10 women and 1 man, with a mean age of 49.8 years (27–83 years). The size of the hemangioma was an average of 23.4 cm (13–35 cm; 8 patients). Five patients had 1 hemangioma, and 6 had multiple hemangiomas. Moreover, the tumors were in both the left and right liver in 6 cases, in only the right liver in 3 cases, and in only the left liver in 2 cases. Eight patients underwent surgical treatment (liver resection: n = 3; liver transplantation: n = 3; enucleation: n = 2); 1 patient underwent embolization; 1 patient took oral prednisone + propranolol; 1 patient underwent radiotherapy. Platelets and coagulation returned to normal in all 8 patients who underwent surgical treatment and in 1 patient who underwent embolization (after 3 interventions). The condition showed no significant improvement in the 2 patients, who received either radiotherapy or prednisone (Table 3).

**Table 3:**
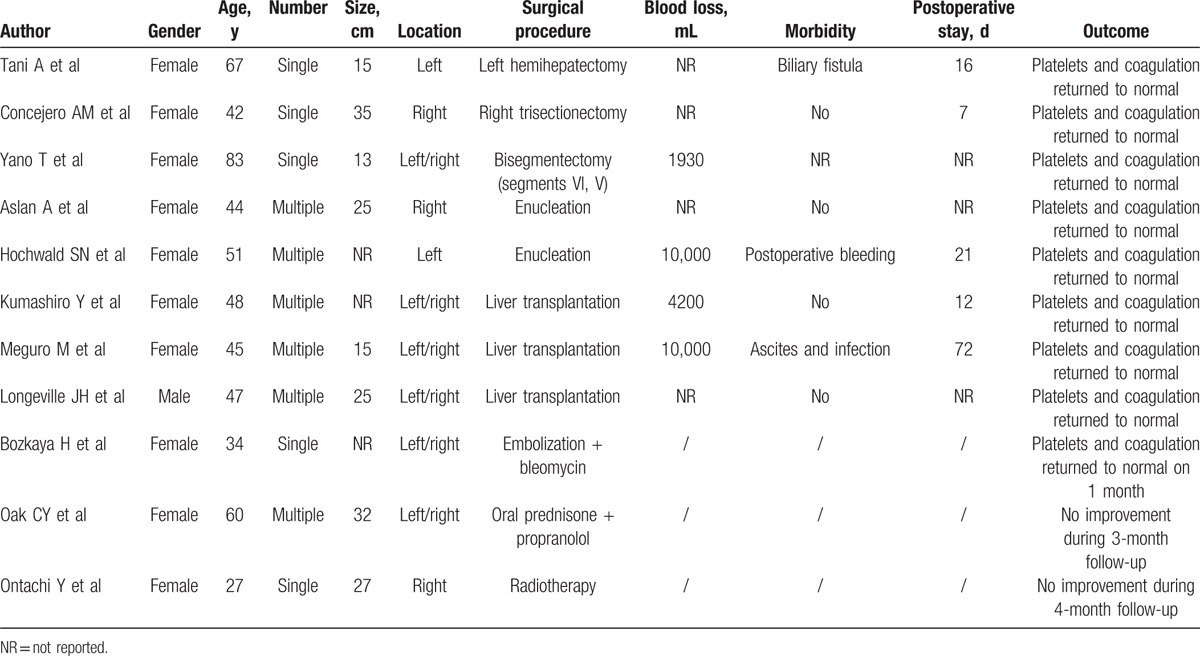
Case reports of Kasabach-Merritt syndrome associated with giant liver hemangioma.

## 4 Discussion

A hemangioma is the most common benign liver tumor, accounting for 73% of all benign liver tumors,^[14]^ with a detection rate of 3% to 20% during autopsy. Hemangiomas are more common in middle-aged women (male:female, 1:5–6).^[15]^ and histologically classified into sclerosing hemangioma, vascular endothelial hemangioma, capillary hemangioma, and cavernous hemangioma, among which the cavernous hemangioma is the most common. Based on tumor size, a hemangioma is classified into 3 types: small (<5 cm), large (5–10 cm), and giant (>10 cm).^[14,16,17]^ Most cases of hemangioma are small hemangiomas, which rarely present any clinical symptoms and are usually found during an ultrasound examination or computed tomography scan. Patients with a giant hemangioma, however, may present abdominal distension and pain, and have coagulation disorders and other serious complications. In 1940, Kasabach and Merritt first reported a rapidly growing skin hemangioma with thrombocytopenic purpura in a newborn baby; since then, hemangioma thrombocytopenia syndrome has been known as Kasabach-Merritt syndrome, which is more common in children and uncommon in adults. We retrieved only 11 articles on this condition (all were case reports) published over the past 20 years. In the present article, we reported 5 such cases, the largest report to date.

For patients with adult Kasabach-Merritt syndrome associated with giant liver hemangioma, intratumor thrombus consumed a large amount of coagulation factors, resulting in coagulation disorders and thrombocytopenia, which was a valid indication for surgical treatment.^[4,7,10,12]^ In this report, all 5 patients had thrombocytopenia, prolonged prothrombin time, and hypofibrinogenemia, as well as varying severities of neutropenia and anemia, indicating a severe impact of the giant hemangioma on the hematologic system and coagulation. This situation increases the difficulty and risk of an operation. In this report, the tumor size of all 5 patients was >20 cm. Among the previously reported 11 cases, the tumor size was reported in 8 cases and was >20 cm in 5 cases.^[4,6,10,12,13]^ No cases of hemangiomas <10 cm have been reported to have Kasabach-Merritt syndrome, suggesting that for hemangiomas, a consumptive coagulation disorder is closely associated with the tumor size.

Currently, the primary treatments for liver hemangioma include liver resection, enucleation, and interventional embolism. For patients with Kasabach-Merritt syndrome associated with liver hemangioma, the tumor is usually extremely giant, posing a significant risk during liver resection and enucleation. Cell saver system was used for all the operations of liver hemangioma in our center and the possibility of blood transfusion during operation was low. However, all the 5 patients required blood transfusion and the highest amount of blood loss was up to 8000 mL. The possible reasons for such huge amount of blood loss were as follows.

1. Kasabach-Merritt syndrome causes coagulation disorders and thrombocytopenia.
2. An extremely giant hemangioma (all 5 cases >20 cm) results in a long operative time.
3. The hemangioma compresses major surrounding vessels (such as the portal vein, hepatic artery, hepatic vein, and inferior vena cava) (Fig. 2) and is prone to uncontrolled severe bleeding during operation.

Hence, liver transplantation has been used to treat Kasabach-Merritt syndrome associated with giant liver hemangioma, with good recovery of coagulation and platelets after transplant.^[8–10]^ However, the donor of liver transplantation is rare, and patients must take long-term immunosuppressive agents after the operation. In this report, there was no mortality and only 1 patient developed a biliary fistula after the operation which was resolved after drainage. Moreover, for all 5 patients, platelets and coagulation returned to normal after the operation. These results suggest that liver resection or enucleation could be safely performed on patients with Kasabach-Merritt syndrome, which had the same effect as liver transplantation.

There was still a debate on the surgical procedures for liver hemangioma; some recommend enucleation, which may reduce blood loss and the loss of normal liver tissue,^[18–20]^ whereas others recommend liver resection.^[21,22]^ For liver hemangioma patients with Kasabach-Merritt syndrome, we recommend liver resection for the following reasons.

1. An extremely giant liver hemangioma (>20 cm) could occupy a large space of the abdomen, making it difficult and dangerous to mobilize the liver. By contrast, after preligation of the unilateral hepatic artery and portal vein, the tumor would become softer and smaller, making it easier to mobilize the liver and, thus, reducing bleeding.
2. In most cases, an extremely giant hemangioma occupied the whole hemiliver or even 3 lobes of the liver, and as a result, liver resection will not lead to a substantial loss of normal liver tissue. For the 5 patients in this report, 4 patients underwent liver resection, and only 1 patient underwent enucleation (the case with the smallest tumor in this report).

According to the literature reports, oral prednisone and radiotherapy are ineffective for liver hemangioma,^[12,13]^ with no significant improvement in coagulation or platelets. One patient underwent embolization, and short-term follow-up showed improvements in platelets and coagulation^[11]^; however, long-term efficacy must be further confirmed because the hemangioma is still present.

In summary, adult Kasabach-Merritt syndrome associated with giant liver hemangioma is uncommon; abnormalities in hematological and coagulative systems can return to normal after operation. For these patients, liver resection may be a better option than enucleation and liver transplantation.
